# The Effects of Bicycle Simulator Training on Anticipatory and Compensatory Postural Control in Older Adults: Study Protocol for a Single-Blind Randomized Controlled Trial

**DOI:** 10.3389/fneur.2020.614664

**Published:** 2021-01-18

**Authors:** Shani Batcir, Omri Lubovsky, Yaacov G. Bachner, Itshak Melzer

**Affiliations:** ^1^Schwartz Movement Analysis & Rehabilitation Laboratory, Department of Physical Therapy, Faculty of Health Sciences, Ben-Gurion University of the Negev, Be'er Sheva, Israel; ^2^Department of Orthopedic Surgery, Barzilai Medical Center, Ashkelon, Israel; ^3^Department of Public Health, Faculty of Health Sciences, Ben-Gurion University, Be'er Sheva, Israel

**Keywords:** falls, aging, balance training intervention, balance control ability, balance reactive response

## Abstract

**Background:** Falls are the leading cause of fatal and non-fatal injuries among older adults. Perturbation-Based-Balance Training (PBBT) is a promising approach to reduce fall rates by improving reactive balance responses. PBBT programs are designed for older adults who are able to stand and walk on a motorized treadmill independently. However, frail older adults, whose fall rates are higher, may not have this ability and they cannot participate. Thus, there is a critical need for innovative perturbation exercise programs to improve reactive balance and reduce the fall risks among older adults in a wider range of functioning. Trunk and arms are highly involved in reactive balance reactions. We aim to investigate whether an alternative PBBT program that provides perturbations during hands-free bicycling in a sitting position, geared to improve trunk and arm reactive responses, can be transferred to reduce fall risks and improve balance function among pre-frail older adults.

**Methods:** In a single-blinded randomized-controlled trial, 68 community-dwelling pre-frail older adults are randomly allocated into two intervention groups. The experimental group receives 24-PBBT sessions over 12-weeks that include self-induced internal and machine-induced external unannounced perturbations of balance during hands-free pedaling on a bicycle-simulator system, in combination with cognitive dual-tasks. The control group receives 24 pedaling sessions over 12-weeks by the same bicycle-simulator system under the same cognitive dual-tasks, but without balance perturbations. Participants' reactive and proactive balance functions and gait function are assessed before and after the 12-week intervention period (e.g., balance reactive responses and strategies, voluntary step execution test, postural stability in upright standing, Berg Balance Test, Six-meter walk test, as well as late life function and fear of falling questionnaires).

**Discussion:** This research addresses two key issues in relation to balance re-training: (1) generalization of balance skills acquired through exposure to postural perturbations in a sitting position investigating the ability of pre-frail older adults to improve reactive and proactive balance responses in standing and walking, and (2) the individualization of perturbation training to older adults' neuromotor capacities in order to optimize training responses and their applicability to real-life challenges.

**Clinical Trial Registration:** www.clinicaltrials.gov, NCT03636672 / BARZI0104; Registered: July 22, 2018; Enrolment of the first participant March: 1, 2019. See Supplementary File.

## Introduction

Older adults' falls and fall-related injuries are serious and costly health problems. Falls are the leading cause of fatal and nonfatal injuries, and it is reported that between 30 and 40% of community-dwelling older persons fall at least once a year (1). Of those who fall, about 30% suffer moderate-to-severe injuries that reduce mobility and decrease independence, and are responsible for 70% of accidental deaths in persons aged 75 and older (2, 3). Ninety-five percent of hip fractures are caused by sideways falls (1, 3) and result from pure medio-lateral balance control. The psychological impact of a fall often results in a self-restricting decrease in activities, along with a decrease in quality of life (4). Apart from the personal suffering, falls constitute a high cost for public healthcare systems worldwide (5). In the US in 2014, the average costs of fatal and non-fatal falls were $26,340 and $9,780, respectively. The estimated direct medical cost for fall-related injuries was $31.3 billion (6).

Balance is an important foundation for independent daily functioning (3, 7, 8), and is a major training component in fall prevention programs (1–4, 9). Ineffective balance compensatory (reactive) reactions following an external-induced unexpected loss of balance such as a push, slip, or trip is one of the major causes of falls in older adults (9). Unexpected loss-of-balance situations trigger reactive balance responses which act to restore equilibrium, these responses depend on the size, type, and direction of the perturbation (10–12). Fixed base-of-support (BoS) strategies (feet remaining in place) are used to restore balance with ankle, hip, trunk, and arm movements during minor-to-moderate unexpected perturbation magnitudes, whereas at larger balance perturbations, change of BoS strategies such as a stepping response are used (9). When balance is unexpectedly lost, a quick reactive step can prevent a fall from occurring (9); however, it was also found that hip, trunk, and arm movements are also part of reactive stepping reactions (9, 12–15). A recent laboratory study of 83 older adults with varying histories of falls who were exposed to a wide range of perturbation magnitudes found that about 61% of these resulted in fixed BoS balance reactions without the need to recover balance by stepping (15). In fact, by using hip, trunk, and arm movements, the older adults were able to decelerate the center of mass (CoM) movement over the BoS, which may result in an ability to perform balance recovery steps at a higher perturbation magnitude, i.e., higher step threshold. A low step threshold was previously shown to be an independent predictor of a future fall (16–21).

Perturbation-based balance training (PBBT), where participants experience repeated postural perturbations during standing or walking in a safe and controlled environment, is a relatively novel approach of fall-prevention exercise that aims to specifically improve reactive balance control in situations where balance is lost unimpededly (22–25). Unlike conventional forms of balance exercise, only a few repetitions of PBBT led to lasting improvements (i.e., 6–12 months) in reactive balance control (26) and even prevented falls in daily life (27). Other studies also demonstrated that older adults who participated in PBBT programs could adapt in a reactive and proactive manner (22, 27–29). Mansfield et al. (22) reported 46% fewer falls than those in the control groups and showed a reduction in diverse risks of falls (22–29) and their incident rate (22–24).

PBBT exercise programs use different mechatronic systems that provide external perturbations in various ways during standing and walking positions. These intervention programs and devices are designed to specifically train the change of BoS strategies (i.e., reactive stepping or grasp reactions) in older adults who are able to stand or walk independently without external support i.e., without holding the handlebars, lasting in a range of 20–45 min each (22–29). Because of the difficulty of the PBBT approach, it may not match frail older adults or people in pre-walking phases of rehabilitative in neurological patients. Therefore, these populations cannot gain from the PBBT that has been conducted to date. In order to match the PBBT approach to a wider range of older adults and patient populations, we aimed to design an alternative PBBT program that uses a new technology that provides unexpected perturbations while sitting, thus, focusing on training reactive and proactive hip, trunk, and arm reactive movements. We were also inspired by recent works that found that older adults who bicycle outdoors regularly have better balance control than aged-match controls (30, 31), and the amount of outdoor bicycling was associated with better balance control (32).

Bicycle training is beneficial for people suffering from heart disease (33), Parkinson's disease (34), and diabetes (35), and it reduces cholesterol, hypertension, and body fat (36), increases muscle power and endurance (36), improves gait parameters (34), executive function (34, 35), and quality of life (33) in older adults. However, using a stationary bicycle as a means of balance control and fall prevention for this population has been found ineffective (37–39). This is not surprising since stationary bicycling does not specifically train balance skills. However, we wondered if the health advantages of bicycling could be combined with the PBBT principals to create a perturbation-based bicycling training program aimed specifically to challenge trunk and arm reactive responses and proactive balance movements in a sitting position to improve reactive and proactive balance in standing and walking in older adults.

In this study we aimed to investigate a new PBBT method and program and to determine whether 12-weeks perturbation-based bicycling training can improve reactive and proactive balance in standing and walking and reduce fall risk in pre-frail older adults, because age-related deterioration of balance function that leads to an increased risk of falling affects all older adults.

**Figure 1:**
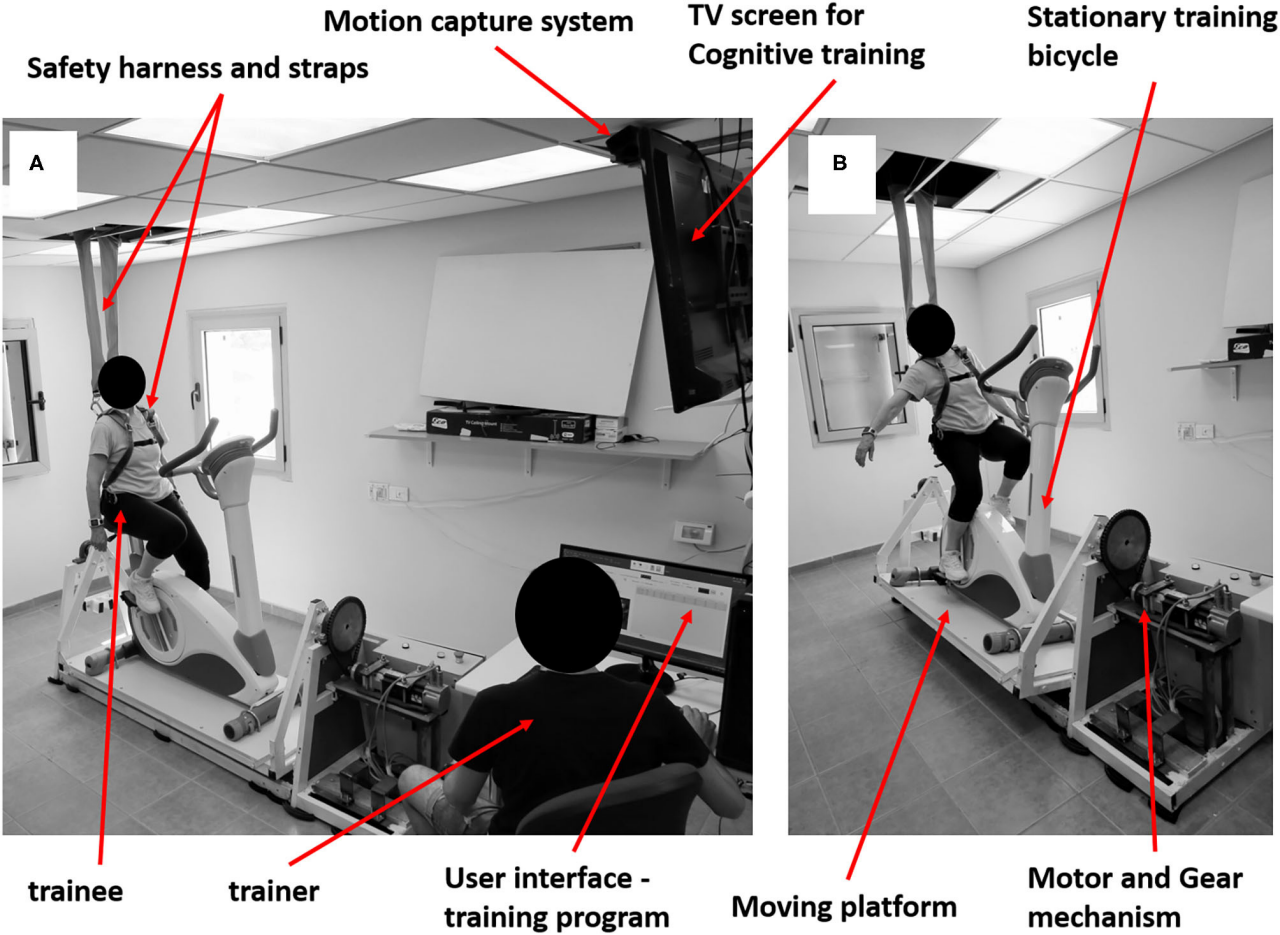
Photos of the PerStBiRo system. The system provides Medio-Lateral tilting perturbations in two ways: self-induced internal **(A)** and external machine-induced **(B)** perturbations. **(A)** Represent a self-induced 'internal' balance perturbation, while the trainee keeps balance when the motor is released, and the moving platform is “floating” during pedaling. **(B)** Represent an external machine-induced programmed balance perturbation during bicycling. The PerStBiRo system is composed of a stationary training bicycle mounted on a moving platform, servo-motor, motor's motion control system, gear mechanism, motion capture system (Microsoft Kinect^KM^ system), safety harness, TV screen and a trainer station with the host PC. The host PC consists the main computer program and serves as a user interface for creating training programs, running them, monitoring upper body movements in real time and analyzing the post-session balance responses.

A new PBBT method called the Perturbation Stationary Bicycle Robotics (PerStBiRo) system was developed especially for this novel training program (Figure 1). The PerStBiRo system is composed of a stationary training bicycle mounted on a moving platform and a motor that provides unexpected medio-lateral (ML) tilting perturbations during hands-free bicycling, aiming specifically to improve reactive and proactive trunk and arm balance reactions. Perturbations are provided in two forms, “internal” and external balance perturbations. The internal perturbations are self-induced and provided by the unfixed and unstable mode of the moving platform, i.e., the platform is “floating” and subjected to the forces exerted on it by the trainee during pedaling (Figure 1A). These situations fall under proactive balance control training. The external perturbations are machine-induced programmed and are provided unexpectedly during bicycling (Figure 1B). These situations fall under reactive balance control training. Our training program, with the help of the PerStBiRo system, is built on a gradual increase in difficulty of several exercise components with respect to motor learning, strength endurance, and especially balance control, such as increasing perturbation magnitudes, varied practice in a block or random perturbation order, and two external cue types (visual or sensorimotor) that are provided to better implement motor learning of balance control and improve the trainee's internal sensorimotor feedback (38). During training, concurrent cognitive visual tasks are provided to distract the participant, thus, facilitating implicit learning and automatization, similar to everyday situations where balance is lost unexpectedly.

We hypothesize that older adults will generalize and transfer the learned reactive trunk and arm movements that will be consistently trained during perturbation-based bicycling training in a sitting position into balance control performances in standing and walking. This research addresses a key question about the generalizability of balance rehabilitation and the underlying locomotor plasticity of older adults using perturbation-based bicycling training as an innovative approach, and the degree to which a perturbation-based bicycling training protocol transfers to laboratory losses of balance—a crucial aspect in clinical application.

## Methods and Analysis

This is a parallel single-blinded randomized-controlled trial (RCT) that follows the recommendations of SPIRIT 2013 (see Table 1 for the study flow diagram). Older adults are randomly assigned to one of two groups: (1) Perturbation Training during hands-free Stationary Bicycling Riding (PerTSBR), or (2) Training of Stationary Bicycle Riding without perturbations (TSBR). Both groups are trained in a sitting position by the same PerStBiRo system (see Figure 1) twice a week for 12 weeks, (i.e., details in regard to the magnitude and progression of the balance training is in the text (experimental and control group interventions) and Table 2. Compensatory (reactive) and anticipatory (proactive) balance control during standing and walking, functional balance, function, fear of falling, and aerobic endurance are measured pre-and post-training at the Schwartz Movement Analysis & Rehabilitation Laboratory in the Physical Therapy Department at the Faculty of Health Sciences at Ben-Gurion University of the Negev, Israel. The intervention is being provided inside participants' community centers or in their protected housing.

**Table 1:** SPIRIT flow diagram of the effects of bicycle simulator training on balance control in older adults.

	**Enrolment**	**Allocation**	**Post-allocation**		
	***–T_**1**_***	**0**	***T_**1**_***	***Training measurement***	***T_**2**_***
**ENROLLMENT**					
**Eligibility screen**	X				
**Informed consent**	X				
**Questionnaires**Demographics, medical history, and past falls	X				
**Allocation**		X			
**INTERVENTIONS**					
***Experimental PerTSBR*** (12 weeks, twice a week)			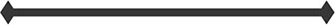		
***Control TSBR*** (12 weeks, twice a week)			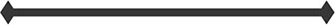		
**ASSESSMENTS**					
**Primary outcome measures**					
***Reactive balance control***—**Compensatory Step Execution tests during standing and walking** (single-step and multiple-step thresholds, first-step recovery initiation duration, first step duration, total balance recovery duration, total CoM displacement).			X		X
**Secondary outcome measures**					
***Balance postural control***—**Postural stability test**(traditional CoP sway and SDA parameters)			X		X
***Proactive balance control***—**Voluntary step execution test** (Reaction Time, Foot Contact Time, Preparation Time, Swing Time)			X		X
***Clinical functional balance***—BBS			X		X
***Aerobic capacity***—6MWT			X		X
**Questionnaires**MMSE, LLFDI, FES-I			X		X
**Performance monitoring during training:** ° Balance recovery (Experimental PerTSBR only) ° Cognitive dual task ° Bicycle resistance				X	

*Overview of time assessments and outcome measures. Pre-study personal interview (-T1); Pre-intervention baseline assessment (T1), Assessment during each of the 24 training sessions during 12-week interventions (Training measurement), Post-test assessment (T2)*.

**Table 2:** Details of the PerTSBR intervention training program–the intensity and the progression levels.

**Training session (number)**	**Platform tilting (deg)**	**Platform peak Vel. (deg/sec)**	**Platform peak Acc. (deg/sec^**2**^)**	**Type of training (blocked/random)**	**Type of perturbations (internal, external)**	**Type of external cues (none, visual, sensorimotor)**	**Number of perturbations per minute**	**Pedaling intensity (bicycle resistance)**	**Cognitive task type and difficulty**
1	0°	Hands-free cycling practice				None	0	0	None
2	2°	10	10	Blocked Lt	External only	None	4	0	None
3	3°	20	20	Blocked Rt	External only	Visual	4	1	None
4	4–5°	25	25	Blocked Rt-Lt	External only	Visual	4	1	None
5	5–7°	30	30	Blocked Rt-Lt	Internal & External	Visual	4	1	None
6	5–8°	30	30	Blocked Rt-Lt	Internal & External	Sensorimotor	4	1	None
7	6–9°	30	30	Random	Internal & External	Sensorimotor	5	1	Find the odd one out of 1
8	5–10°	30	30	Random	Internal & External	Sensorimotor	5	1	Find the odd one out of 2
9	7–11°	30	30	Random	Internal & External	Sensorimotor	5	2	Find the odd one out of 3
10	8–12°	30	30	Random	Internal & External	Sensorimotor	5	2	Find a specific object 1
11	5–13°	30	30	Random	Internal & External	Sensorimotor	5	2	Find a specific object 2
12	5–13°	30	30	Random	Internal & External	None	6	2	Find the differences 1
13	9–14°	30	30	Random	Internal & External	Sensorimotor	5	2	Find the differences 1
14	10–15°	30	30	Random	Internal & External	Sensorimotor	5	2	Find the differences 2
15	5–16°	30	30	Random	Internal & External	Sensorimotor	5	2	Find the differences 2
16	5–16°	30	30	Random	Internal & External	None	6	3	Find the differences 3
17	11–17°	30	30	Random	Internal & External	Sensorimotor	5	3	Find the differences 3
18	12–18°	30	30	Random	Internal & External	Sensorimotor	5	3	Find the differences 4
19	5–19°	30	30	Random	Internal & External	Sensorimotor	5	3	Find the differences 4
20	5–19°	30	30	Random	Internal & External	None	6	3	Find the differences 5
21	13–20°	30	30	Random	Internal & External	Sensorimotor	5	3	Find the differences 5
22	14–20°	30	30	Random	Internal & External	Sensorimotor	5	3	Find the differences 5

Details of the external perturbations, cognitive tasks, and bicycle resistance during the 22 potential training sessions. Deg, degrees; sec, seconds; sec^2^, second ^*^ second; Vel, velocity; Acc, acceleration.

### Participants

A convenience sample of 68 community-dwelling older adults will be recruited (see sample size estimation below). Eligibility criteria are: 70 years of age or older, ability to walk independently without assistive devices, independence in daily living activities, and provision of a medical waiver signed by their primary care physician allowing participation in physical training that requires pedaling on a stationary bicycle two or three times a week. After completing a medical history questionnaire via an in-person interview at enrollment, volunteers are excluded if they suffer from: (a) ischemic heart disease which restricts exercise, (b) chronic obstructive pulmonary disease, (c) uncontrolled blood pressure, (d) severe vision problems (blindness), or (e) cognitive impairment, scoring <24 on the Mini-Mental State Examination. Further exclusion criteria include: (f) a period <1 year after hip or knee replacement surgery or after fractures of the lower extremities, (g) amputation of a lower limb, (h) neurological diseases or after a stroke, and (i) inability to ambulate independently, and/or (j) weight > 120 kg (exceeds safety harness weight limits). The study was approved by the Helsinki ethics committee at Barzilai Medical Center, Ashkelon, Israel (BARZI0104).

### Recruitment, Randomization, Group Allocation, and Blinding

Participants are recruited using advertisements, flyers, and personal contacts from community-dwelling centers and from several protected housing institutes in Beer-Sheva, Israel. They are assigned using randomization to one of the two intervention groups (PerTSBR or TSBR). The random allocation sequence is computer generated, and blocked randomization will ensure equal numbers allocated to each group. Group allocation is performed centrally by the principal investigator, who is involved in recruiting, scoring assessments, or administering the interventions (i.e., concealed allocation). Outcome measures will be obtained by two research assistants who are blinded to group allocation. Participants are not fully blinded to group allocation since they train on the same mechatronic device but without perturbations.

### Informed Consent

When an older adult is willing to participate in the study, a researcher approaches the subject, schedules a pre-study meeting to explain the study, and provides the study information sheet, information form for the primary care physician, and the permission to participate in the study/consent form. Participants are informed that this is a novel intervention method of a technology that aims to improve balance while riding a new stationary bicycle device, and that we hypothesize that they most likely will improve their function. In addition, they will be informed that both groups will perform bicycle training with or without perturbations thus, both are expected to improve function. To maintain the motivation of older adults to participate, they will be offered to participate in “other” exercise program after the training period. They are also informed that the training might be difficult in the beginning; thus, some muscle soreness will occur, and that participants are free to withdraw from the study at any time point, without consequence. Participants may also be withdrawn from the study due to changes in their health status that affect eligibility. A researcher will answer the participant's questions about the study, and participants may discuss the study with their family members, friends, or healthcare providers. All participants sign an informed consent statement via personal interview at enrollment. The informed consent process is documented by research personnel.

### The Interventions

**Figure 2:**
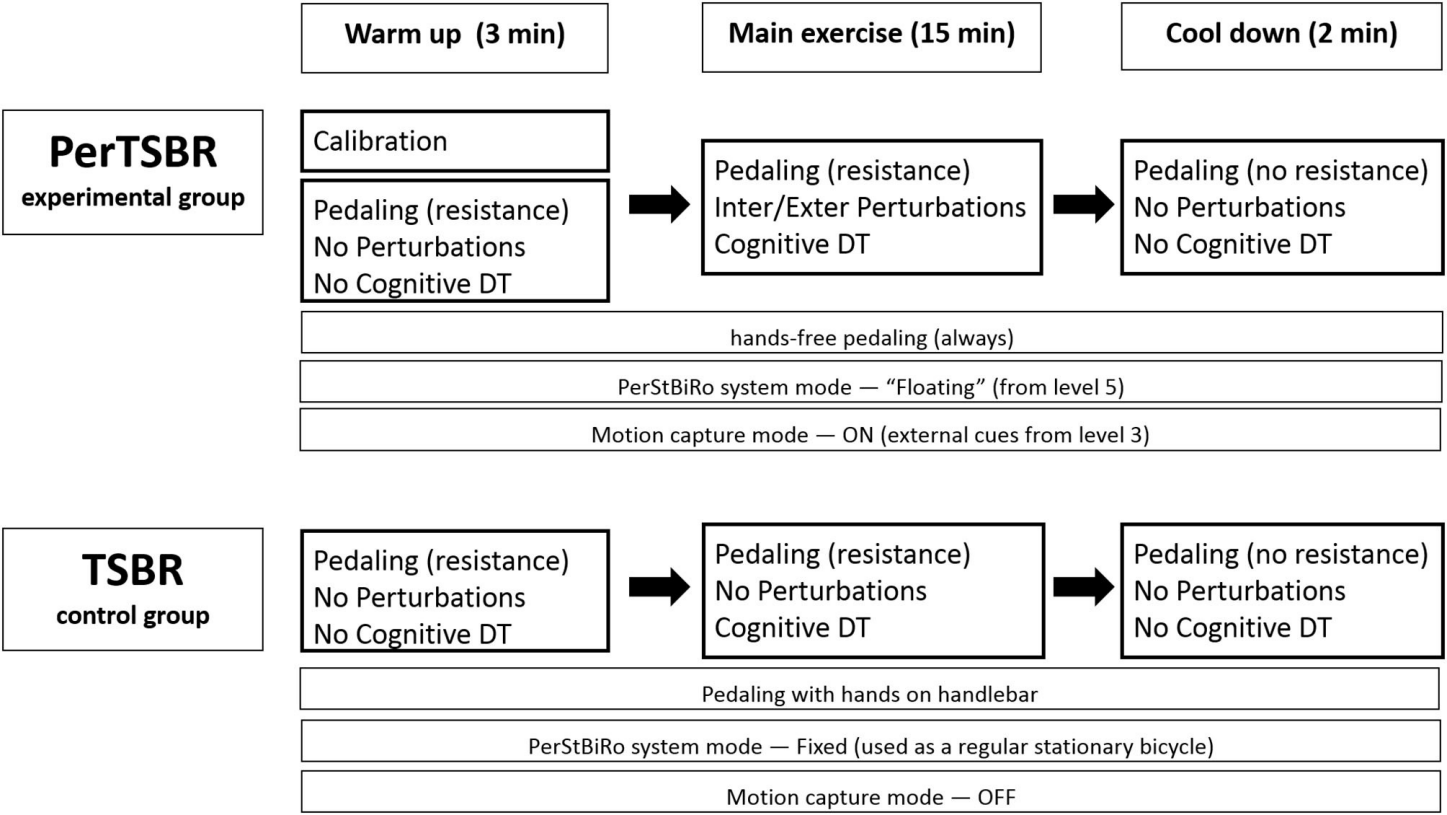
Training session templates for PerTSBR and TSBR groups. Resistance= stationary bicycle resistance while pedaling, DT=dual tasks, Inter/Exter= internal/external, “floating” = a PerStBiRo–system's mode in which the moving platform and the stationary bicycle are unfixed and unstable; thus, it is subjected to tilting by the forces acting on it. Fixed mode is when the PerStBiRo is locked vertically, and it is used as a regular stationary bicycle. Motion capture mode refers to whether the motion capture system i.e., Microsoft Kinect^KM^ system works and monitors the trainee's movements, providing them with real-time external cues of their balance reactions. Levels refer to the level of the training program (details in Table 1).

Trained physiotherapists conduct individualized training. They received training to operate the PerStBiRo system (Figure 1), and experienced it themselves to be able to determine the challenge for a participant and adjust the difficulty of the training. We use the same PerStBiRo system to train both, the PerTSBR and TSBR groups. Each group receiving 24 training sessions, twice a week for 12 weeks. Based on a recent systematic review (24) of PBBT paradigms, programs consist of low perturbation magnitudes as this is a more feasible program for each older adult, requiring longer training periods for significant improvement of reactive balance responses and reduction in fall incidence (24). Increasing the total PBBT volume increases the likelihood of its effectiveness (40). Since our PBBT program focuses on improving the trunk and arm balance recovery reactions in a sitting position, the program duration is as applied at lower magnitudes of PBBT during standing or walking, intensities that usually evoke fixed BoS strategies. Each session lasts for 20 min and includes three parts (Figure 2): stage (1) warm-up-−3 min of self-paced pedaling with the same bicycle resistance for both groups, corresponding to the training program level, without perturbations and cognitive tasks; stage (2) main exercises−15 min of perturbation training during hands-free stationary bicycling (PerTSBR). For details, see “Experimental group intervention,” with perturbations and “Control group intervention” without perturbations (TSBR) in combination with concurrent visual cognitive tasks for both groups. The graduated difficulty levels in bicycle resistance and the cognitive tasks are the same for both groups and determined according to the training program (details in Table 2); and stage (3) cool down-−2 min of self-paced pedaling without bicycle resistance, without perturbations and cognitive tasks. During stage 2 the participants are instructed to “ride the bicycle at your preferred pace and try to stabilize yourself. Try also to do your best in the cognitive tasks.” The cognitive dual-task exercises are presented using Microsoft Power Point on a TV screen 2.5 meters in front of the pedaling trainee's head. It includes tasks such as “Find the odd one out,” “Find a specific object in the picture,” and “Find the differences” between two pictures. For all tasks presented, the participant must state out loud where the object is, then have this checked by the trainer, and if the answer is correct, the next task is presented. During the training sessions trainees wear a loose safety harness that can arrest falling using the PerStBiRo system, but still allow comfortable pedaling and execution of balance recovery reactions with the upper body without the harness suspension. In both groups, each participants activities are documented in each session. See Figure 2 for a single-training session template and see detailed explanations in “Experimental intervention” and “Control intervention.”

### Experimental Group Intervention

**Figure 3:**
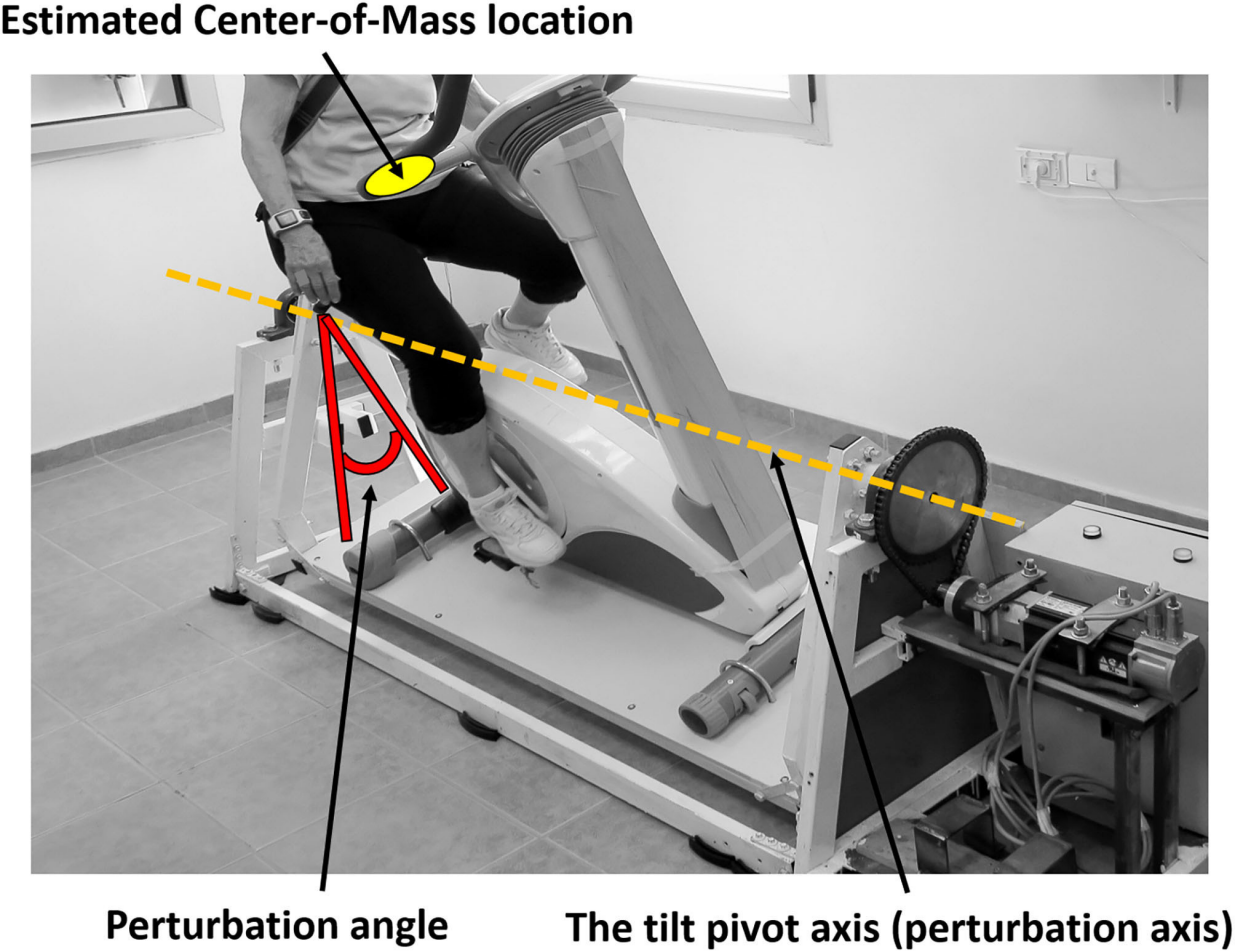
Medio-lateral perturbation system to evoke reactive trunk and upper body balance reactions.

The PerTSBR experimental group participants receives a combination of internal and external ML tilting perturbations during hands-free bicycling under dual-task conditions. This is provided by roll-angle (tilt) balance perturbations that aim to evoke trunk and arm balance recovery responses. The tilt pivot axis is formed by two ball bearings in the front and rear of the moving platform, crossing along the training bicycle and passing under the trainee's seat position (see Figure 3). The tilt pivot axis is lower than the trainee's CoM that is located in the pelvis above the bicycle seat. Therefore, when tilting the trainee's CoM aside rapidly, the trainee is forced to respond reactively with trunk and arm movements. The PerStBiRo system provides a maximum right and left perturbation tilt angle of 20° (each side) with maximum acceleration and deceleration of 30 m/s^2^ and maximum velocity of 30 m/s, which is usually that of a triangular motion profile for each perturbation (acceleration—deceleration). The computer program allows the therapist trainer to determine the perturbation training plan and control all balance exercise parameters: maximum acceleration/deceleration, maximum velocity, angle of perturbation, the number of right/left perturbation repetitions, and the delay time between each pair of consecutive perturbations.

**Figure 4:**
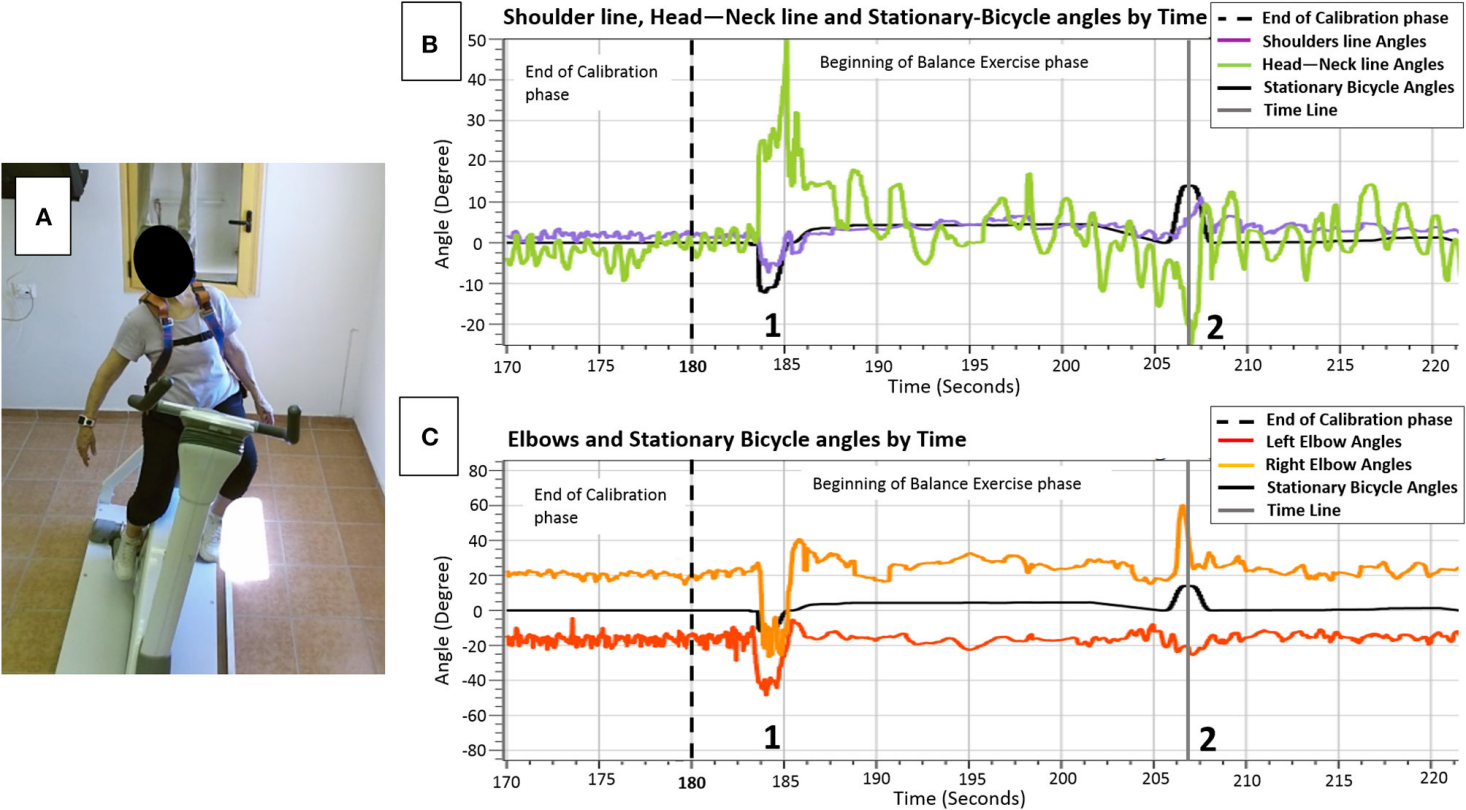
**(A)** sample of upper body movements (horizontal colored lines) and the PerStBiRo system's stationary bicycle (horizontal black lines) by time during the calibration phase (left of the dotted line in **B,C)** and during balance perturbation exercise phase, focusing on the upper body reactive balance response of an 82-year-old trainee following a programmed 20° right tilting perturbation **(A** and gray timelines in **B,C)**. **Shoulder line angle**—the angle of the participant's shoulder line and the ground (**B**, horizontal purple line); **Head–Neck angle**—the angle of the participant's head-to-neck line and the vertical line to the ground (**B**, horizontal green line). Points 1 + 2 indicate external perturbations that lead to sharp and large upper body balance reactions. The time range 1–2 indicates internal perturbations, the upper body oscillations when riding on an unstable surface—as seen the horizontal black lines, that represent the stationary bicycle angles, are not exactly on the vertical 0° position.

Diagnosis of an effective balance response to each trainee following unannounced balance perturbation is the basis for providing the trainee with real-time customized sensorimotor cues. These cues will lead to improvement in the trainee's internal sensorimotor feedback of successful trunk and arm balance recovery reactions and, therefore, to better implementation of motor learning of balance reactive control (41). Thus, a calibration phase is needed. During the calibration phase (Figures 4B,C, left of the black dotted lines), by capturing the upper body joints, the computer program calculates two angles: (1) the **shoulder line angle**—the angle of the participant's shoulder line and the ground (Figure 4B, horizontal purple line), and (2) the **head–neck angle**—the angle of the participant's head to neck line and the vertical line to the ground (Figure 4B, horizontal green line). The sequence of angles that maintains more stability and less noisy singles are automatically selected as the key-factor angles that the program relies on in determining the trainee's sitting posture and differentiating upper-body oscillations during pedaling (Figure 4B, calibration phase, horizontal purple and green lines) from significant reactive balance responses following external perturbations later into the training session (Figure 4B, balance exercise phase, horizontal purple and green lines at points where the horizontal black line shows humps). Then, relying on this key-factor angle, the real-time sensorimotor cue to an effective balance reaction is given by returning the PerStBiRo system's training bicycle to its vertical position. For example, in Figure 4, the trainee was exposed to a programmed 20° right tilting perturbation at the 206th second of the training session, indicated by the gray timeline in Figures 4B,C. However, an effective sharp and large upper body balance response (Figure 4A) had already been identified when the PerStBiRo system's training bicycle was at about 14° (Figure 4B, the hump on the horizontal black line); thus, the perturbation was immediately stopped, and the device was returned to its vertical position. In addition, the elbow angles are also recorded for monitoring arm reactions (Figure 4C).

All perturbation parameters, as well as the trainee's shoulder line angles, head–neck angle, and elbow angles are displayed on the host-PC screen in real time. Also, the angle of perturbation as soon as an effective balance response is detected by the Microsoft Kinect^KM^ system is displayed in real time. Thus, the therapists can compare the programmed perturbation angle with the actual perturbation angle (once an effective balance response is detected) and monitor the patient's ability to recover from perturbations along the training session.

During the first stage of the training session, i.e., the warm-up phase, the PerStBiRo system calibrates the trainee's upper body movements and their body configuration and position (see Figure 4). The customized calibration is performed in the same fixed mode or unfixed and unstable “floating” mode as the PerStBiRo system is expected to be in during a specific training. Fixed mode is when the PerStBiRo system is locked vertically and used as a regular stationary bicycle, while the “floating” mode is when the moving platform is unfixed and unstable, floating like a surfboard and is subjected to the forces acting upon it by the pedaling trainee. Calibration is necessary to identify an effective reactive balance response later in the training session.

In the second stage of the training session, i.e., the main training phase, has internal and unannounced external balance perturbation exercises in self-paced hands-free pedaling under dual-task conditions. Pedaling the PerStBiRo system in its unfixed and unstable “floating” mode causes self-induced perturbations similar to outdoor bicycling, and challenges proactive balance control. These internal perturbations are initiated from training level 5 (see Table 1). The programmed unannounced external perturbations are ranged from low to high controlled, unexpected machine-induced ML tilting perturbations, which evoke fast upper body reactive balance responses (i.e., trunk, hip, head, and arm movements). These perturbations can be programmed and delivered as a block or random (in onset, magnitude, and direction) type of training. During perturbation exercise, the PerStBiRo system provides trainees with real-time visual or sensorimotor external cues (cues are given from training level 3—see Table 1). (1) A visual cue for a beginner trainee is obtained by self-watching their balance performance in real-time on the television (TV) screen, like a mirror view. (2) For an advanced trainee, a sensorimotor cue is obtained by giving a real-time sensorimotor cue to the trainee's balance reaction following a perturbation. Once an unexpected balance perturbation is given, when an appropriate balance reaction is detected, the tilting platform (the perturbation) is stopped immediately, and the PerStBiRo system returns to its vertical position (details under “Perturbations”). This sensorimotor cue will lead to improve the trainee's internal sensorimotor feedback, therefore, to better implement motor learning of reactive balance control (41). In addition, concurrent visual cognitive tasks are displayed on the PerStBiRo system's TV screen.

The third stage of the training session, i.e., the cool-down part, consists of self-paced pedaling without bicycle resistance, and without cognitive tasks and external programmed perturbations. However, the fixed or “floating” PerStBiRo system mode remains as it was during a specific training, so the trainee may be exposed to self-induced internal perturbations.

The difficulty of the perturbation training level is adjusted according to the trainee abilities, starting from the lowest level at the first training session. If the trainee is able to recover from all perturbations during the session (i.e., one did not hold the bicycle handlebars or fall into harness system during the session and feels that they can be further challenged), a higher level of perturbations will be introduced in the next session. In case the trainee was not able to recover, the same level of perturbations is introduced again until the participant can successfully recover balance in the entire session. Assistance and support are provided for trainee who feel uncomfortable in the initial phase of the training. However, they are encouraged to perform exercises with no or minimal external support.

### The Perturbation Training Program and Progress

The training program (Table 2) consists of 22 potential training levels, which contain a gradual increase in difficulty in several exercise components with respect to motor learning, strength, endurance, and especially balance control:

1) The training always starts with hands-free cycling practice to avoid external support on the handlebars that significantly reduces the postural responses (42) and to calibrate the software to identify balance recovery responses.

2) The perturbation magnitude increases (i.e., increases displacement, velocity, and accelerations of the tilting translations).

3) The type of training shifts from the block PBBT method at the beginning, where the participant is aware of the direction of perturbation (right–left–right etc'), with a similar time interval between perturbation, thus these are an expected perturbation. Than announced random PBBT was introduced (random in onset, direction, and tilting magnitude of the perturbation). Varied practice in a random order results in better motor learning (43). This is supported by a recent study that found that, in retention and transfer tests, the results indicated higher values for random than for block perturbation training in healthy young participants (44).

4) The type of perturbation begins with external perturbations only when the simulator device is fixed and stable in the first sessions and changes to a combination of external and internal perturbations that are the unfixed and unstable surface which is affected by the forces exerted on the device while the trainee pedals in increasing intensity. Practicing in a variable and unstable environment is an advanced practice that contributes to improve skill acquisition (45).

5) The external cue type also changes from no cue at all to a visual cue that is like real-time viewing of a mirror on a TV screen during exercise and then changing to an external sensorimotor cue that leads to improving the intrinsic sensorimotor feedback. Once an unexpected balance perturbation is given, when an appropriate balance reaction is detected, the tilting perturbation is stopped, and the simulator system returns to its vertical position. This intrinsic task feedback provides the learner (trainee) with an implicit cue for successful balance response and provides the best possible motor learning implementation (41). In addition, at training levels 12, 16, and 20, no external cue is given in order to maximize the subject's upper body movements to improve their upper body range of motion (especially of the trunk).

6) The pedaling intensity.

7) The cognitive tasks difficulties also increase along the training process.

### Control Group Intervention

The control group (TSBR) receives 20 min of bicycle riding on the PerStBiRo system in its fixed mode along all of the training program (used as a regular stationary bicycle) without any internal or external perturbations (Figure 2), but dual-task training is provided. Pedaling intensity and cognitive tasks are the same as in the intervention group and follow the levels of difficulty that are reported in the training program (Table 2). This training method was chosen in order to match all other training components (i.e., session time and training period, and pedaling and cognitive demands), except for the balance challenge. Bicycle training programs have been shown to improve trainees' gait parameters (34) and increase muscle power and endurance (36), executive function (34, 35), and quality of life (33) in older adults. The control group not only receives balance, but also other important training components.

### Data Collection and Outcome Measures

First, by personal interview with one of the researchers at the enrollment session (T1), each participant completes demographic, medical history, and past fall questionnaires (see Table 1). Then they undergo two assessment sessions: first at entry of the study (baseline assessment, T1), and the second at the end of the 12-week intervention period (T2). To ensure data quality, two research assessors blinded to group allocation and the training process receive training regarding data collection from the principal investigator and perform all measurements.

### Primary Outcome Measures

Because this study deals with the ability to learn and generalize reactive balance responses that are acquired in a sitting position into a target context of fall risk factors and balance control performances in standing and walking situations, our primary outcome measures to evaluate the effect of PBBT during bicycle riding are derived from the ability to recover from unexpected external perturbations in the compensatory step execution tests during standing and walking. The best observational and kinematic measures to represent the reactive (compensatory) balance responses are: the single-step and multiple-step thresholds (observational parameters), and the first-step recovery initiation duration, first step duration, total balance recovery duration, and total CoM displacement (kinematic parameters) (14). All of these parameters have been previously found to be able to distinguish between younger and older adults (14) and fallers and non-fallers (15). The single-step threshold was previously shown to be an independent predictor of a future fall (16–21) and to reflect unsteadiness in reactive balance control among older adults with varying histories of falls (15). Moreover, excellent inter-observer reliability has been reported for single-step and multiple-step thresholds (ICC_2, 1_ = 0.978 and ICC_2, 1_ = 0.971, respectively; *p* < 0.001), and for first-step recovery initiation duration, first step duration, total balance recovery duration, and total CoM displacement (ICC_2, 1_=0.917, ICC_2, 1_=0.975, ICC_2, 1_=0.950, and ICC_2, 1_=0.918, respectively; *p* < 0.001) (14).

The compensatory step execution test examines reactive balance responses following unexpected platform translations. Perturbations are induced through a mechatronic device that provides controlled and unexpected AP and ML platform surface translations during standing and waking (46). For standing, participants are instructed to stand with feet placed together (heels and toes touching), resting arms naturally at their sides. For walking, participants are instructed to walk at their preferred treadmill walking speed with their hands free to swing (no handrails on the treadmill). Perturbations are synchronized to the initial contact phase of the gait cycle using the three-dimensional (3D) Vicon Motion System (Oxford, UK) and a code written in Mathlab for calculating the subject's own treadmill speed and step width. In standing as well as walking, participants are exposed to random of onset and random right/left/forward/backward unannounced platform translations that are increased systematically and controlled at ten perturbation magnitudes in increasing levels of difficulty. Four directional perturbations (right/left/forward/backward) occur in a random order at each level for a total of 40 perturbation trials (see Table 3 for perturbation details). During examination, participants wear a safety harness that prevents falls, but does not otherwise restrict their recovery balance movements. Falls during the assessment session are defined, as load cell sensors detect 30% or more of body weight suspended by the safety harness (48). Participants wear their own walking shoes and are instructed to react naturally to keep their balance and to prevent themselves from falling in response to perturbations. The increase in perturbation difficulty is adjusted by the examiner according to each subject's ability to recover. In case the subject should fall, grasp the examiner's hand, or ask to stop the test, the examination is stopped and will not continue to the next level. A seated rest break is given whenever needed. The data are analyzed observationally and kinematically for each trail.

**Table 3:** Magnitude of the perturbation i.e., the parameters during the examination protocol of balance reactive response in standing.

**Perturbation size**	**Platform displacement (cm)**	**Perturbations velocity (m/s)**	**Perturbations acceleration (m/s^**2**^)**	**Perturbations random order**
Level 1	3	0.06	0.09	B, R, F, L
Level 2	4	0.06	0.18	B, F, R, L
Level 3	5	0.11	0.35	L, B, F, R
Level 4	6	0.16	0.52	F, R, B, L
Level 5	7	0.22	0.7	B, F, R, L
Level 6	8	0.33	1.1	B, F, L, R
Level 7	9	0.44	1.5	B, R, F, L
Level 8	12	0.66	2.0	B, F, R, L
Level 9	15	0.88	2.5	R, B, F, L
Level 10	18	1.20	3.0	B, R, L, F

*Perturbations will be induced through a mechatronic device that provides controlled and unexpected AP and ML platform translations during standing (47): four directional perturbations (right=R/left=L/forward=F/backward=B) in a random order in each of the 10 levels for a total of 40 perturbations*.

#### Observational Analysis

We verify single-step and multiple-step threshold levels for AP and ML directions following a loss of balance using a Vicon Motion System (Oxford, UK), allowing image pauses, slow motion, and running of the image back and forth. The single-step threshold level is defined as the minimum perturbation magnitude that consistently elicited a single compensatory step for at least two consecutive perturbation magnitudes. The multiple-step threshold is defined as the minimum perturbation magnitude that consistently elicited a sequence of recovery steps. During the analysis, we explore the step recovery strategies during all single-step and multiple-step trials during the whole protocol.

#### Kinematic Analysis

3D kinematic data are collected through optical motion capture (Vicon Motion Systems, Oxford, UK), providing kinematic analysis of a motion sequence. Sixteen infrared cameras cover the lab space, mounted at a height of 2.6 ± 0.2 meters and providing a capture volume of 5.5 × 1.2 × 2.0 m^3^ evenly scattered approximately four meters around the treadmill. The cameras operate and sample simultaneously, at a frequency of 200 Hz, the location of 39 reflective markers placed on anatomical landmarks of the body and another two on the moving platform. The markers are attached to a prepared whole-body flexible suit that comes in several sizes to properly fit each subject. Views from the 16 cameras are mapped onto a 3D coordinate system by the computer (Vicon Systems software) using an internal direct linear transformation algorithm. Perturbations that are followed by execution of a compensatory step (change in BoS) are digitized, transformed, and smoothed using a low-pass filter (Butterworth second-order forward and backward passes) with a cut-off frequency of 5 Hz. The Vicon system was shown to be valid and reliable. Overall trueness (systematic deviations) of a dynamic reference object was −0.23 ± 0.35 mm (−0.24 ± 0.36%) and overall uncertainty (random deviations) for dynamic measurements was 1.11 ± 0.94 mm (1.16 ± 0.99%). For lower body assessment (10 cameras, foot region) during walking, the mean trueness and uncertainty was 0.08 and 0.33 mm, respectively (47).

The following kinematic parameters are extracted: (1) the step initiation duration in milliseconds (ms) is calculated as the time from surface horizontal translation to step initiation, (2) first recovery stepping duration (ms) is calculated as the time from surface translation to foot-contact on the ground, (3) first compensatory step length is calculated as the Euclidian distance in centimeters (cm) that the ankle markers are displaced from step initiation to first step recovery, and (4) Margin of Stability (MoS), which is the distance between a velocity adjusted or “extrapolated” position of the CoM (XcoM) and the edge of an individual's BoS at any given instant in time. The MoS is directly related to the impulse (I) required to cause instability (49). When a participant takes extra steps to recover balance, i.e., a multiple-step reaction, the following parameters are determined: total balance recovery duration (ms), which is the time from surface translation to foot contact on the ground, completing multiple steps to recover balance; recovery step path length, which is the Euclidian distance in cm that the ankle markers are displaced from step initiation to foot contact on the ground, completing full balance recovery; and total CoM path displacement (cm), defined as the distance in cm of the CoM traveled from the initial point prior to stepping to the point where participants completed their full balance recovery. Using this method, Batcir et al. (14) found excellent inter-observer reliability for observational and kinematic analysis.

### Secondary Outcome Measures

Secondary outcomes include balance and aerobic endurance measures that are assessed pre- (T1) and post-(T2) intervention by the following tests:

1) The **Postural Stability Test** measures balance postural control by the body sway in upright standing. The participants are instructed to stand barefoot as still as possible on a force platform in a standardized stance, their feet close together with their hands crossed behind their back (50). Five 30-s eyes-open (EO) and eyes-closed (EC) assessments are conducted for each participant. CoP and ground reaction force data are collected at a frequency of 100 Hz with a single Kistler 9,287 force platform (Kistler Instrument Corp., Winterthur, Switzerland). Balance control is evaluated using both the traditional measures of CoP postural sway (e.g., ML sway range, AP sway range, mean sway velocity, and mean sway area), and the Stabilogram-Diffusion Analysis (SDA) parameters for ML and AP directions: Critical time (Ct), Critical displacement (Cd), and the short-term (Ds) and long-term (Dl) effective diffusion coefficients. This was described in detail by Collins and De Luca (51, 52). The data are extracted from the CoP trajectories using a code written in MatLab (Math Works Inc., Cambridge, MA, USA). The ICC for the CoP sway parameters are excellent in the EC condition: ML sway (ICC=0.933), AP sway (ICC=0.946), sway area (ICC=0.710), sway length (ICC=0.945) (53), and fair-to-good reliability to the SDA parameters: Ds (ICC=0.79), Dl (ICC=0.50), Cd (ICC=0.66), Ct (ICC=0.63) (54). In the narrow-base standing condition, the ML sway range and the mean sway velocity (55), as well as the Cd and Ds (56) are predictive variables for falls (55, 56) and the severity of fall-related injury (50).

2) The **Voluntary Step Execution test** measures anticipatory (proactive) balance control by the ability to perform a quick step. Participants stand on a single force platform with their feet abducted 10° and their heels separated medio-laterally by 6 cm. They are instructed to voluntarily step (50–60 cm long) as quickly as possible following a somatosensory cue given randomly on **one** of their heels (57, 58). CoP movement and ground reaction force data are collected from the Kistler 9287 force platform. A total of **eight** trials are conducted in single-task and dual-task conditions (while performing the modified Stroop task): four forward and four sideways. The average result across task conditions are used for statistical analysis. Participants view an “X” displayed on a screen in front of them. Specific temporal events are extracted from the step execution data: (a) reaction time; (b) foot contact time; (c) preparation time; and (d) swing time (57, 58). The ICC values for intra-tester reliability for older adults are good to excellent for all the step parameters across all task conditions (0.62–0.88) except for the swing time variable (0.32–0.64) (59). The foot contact time and the initiation phase were able to predict future falls (60) and injury from fall (58).

3) The **Berg Balance Scale** (BBS) is a clinical functional balance instrument that assesses balance and fall risk. It is a 14-item performance-based measure of functional balance skills. Each task is scored 0–4 on an ordinal scale, and the total score ranges are 0–56. A higher score represents better balance (61). It highly correlates with other functional measurements. A score <45 indicates individuals at greater risk of falling (62).

4) The **Six-Minute Walk Test** (6MWT) is a sub-maximal test of aerobic capacity measuring the maximum distance that a person can walk in 6 min (63). We use this measure to control for the endurance component of the pedaling training.

5) The **Late Life Function and Disability Instrument** (LLFDI) is a self-reported function measuring difficulty in performing basic and advanced daily physical tasks (64).

6) The **Falls Efficacy Scale-International** (FES-I) evaluates fear of falling while performing indoor and outdoor social and physical daily activities (65).

#### Assessing Upper Body Balance Reactions During Training

During each training session of the experimental PerTSBR group, the data of skeleton joint identification that was obtained by the Microsoft Kinect^KM^ camera is recorded for post-training analysis. Trunk and upper body balance recovery responses are assessed for each of the perturbations by the physiotherapist/trainer using kinematic graphs and a movie of the trainee riding the PerStBiRo system. This data helps the physiotherapist to analyze the trainee's balance behavior during the perturbations and to determine how to proceed with the training program in the next sessions (see Figure 3).

### Data Management

At the baseline assessment (T1), each participant receives a computer-generated personal identification code. All data are collected and stored anonymously with this personal code, and the data are the responsibility of the principal investigator (IM). Participants' medical and personal information obtained in this study are considered confidential, and disclosure to third parties prohibited. Electronic data are stored on secure institutional servers immediately after both assessment sessions. Hard copies of files are stored in locked cabinets in our laboratory. Each participant has an electronic folder, as well as a hard-copy folder with their own personal code consisting of raw and analyzed data, test results, and questionnaires from pre- and post-training assessments. The names and phone numbers of participants are only be accessible to the research team for contact purposes. To ensure protection of participants' personal information, the data collected during the study will be kept confidential, and saved and stored on secure servers for 10 years, are password protected, and will not be shared with anyone outside of the study unless required by law. The data will be deleted from the servers after 10 years. Hard files containing de-identified data and consent forms are stored separately from other data and in locked cabinets in our laboratory. Only researchers from the research team allowed access to the data for the purpose of this study are provided with the password to the file containing identifiers and/or the keys to the locked cabinet/office.

### Sample Size Estimation

Sample size requirements were calculated using PS power calculation software (66). Because the aim of the study is to examine the effect of perturbation-based bicycle training on various aspects of balance control, well-known parameters that reflect reactive, proactive, and standing postural balance control were selected. Calculations were performed separately to determine sample size requirements based on a single-step threshold in standing (reactive balance), voluntary step execution (i.e., foot contact time) (proactive balance), and ML postural sway (standing postural control), all reflective of unsteadiness in balance control between older and younger adults (13, 14, 50, 55, 58, 67, 68) and between fallers and non-fallers (13, 15–21, 55–58, 60). Type I error probability of 0.05 and Type II error probability of 0.2 was applied for the three two-sided test calculations. Based on our preliminary results, the compensatory single-step threshold in standing was 10.8 cm of lateral translation in older adults with bicycle riding habits compared to 7.9 cm in age- and gender-matched controls [data in processing], the ML postural sway in EO was 30.6 mm in older adults with bicycle riding habits compared to 38.8 mm in controls (31), and the step execution times (i.e., foot contact time) in single-task condition of older bicyclists were 104 ms less than those of the controls (921 vs. 1,025 ms) (30). Using net reduction values (2.9 cm, 8.2 mm, and 104 ms, respectively) in combination with the initial variance estimates (standard deviations of 3.45 cm, 7.8 mm, and 134 ms, respectively), it was determined that 27 participants per group would be required. To account for reported attrition rates of about 25% in studies involving older adults (69), we decided to include about 34 participants in each group for a total of 54 (27 × 1.25 = 34).

### Statistical Analysis

PASW Statistics version 26.0 are used for statistical calculations. Baseline characteristics are compared using independent *t*-tests and Mann–Whitney *U*-tests for continuous and ordinal variables, respectively. Descriptive data analysis and tests for the assumptions of normality (Shapiro-Wilk's statistic) are followed by a two-way repeated measure analysis of variance (ANOVA) for within subjects (pre- vs. post-tests) and between groups (experimental vs. control groups). The primary outcome variables are the single-step and multiple-step thresholds, the first-step recovery initiation duration, first step duration, total balance recovery duration, and the MoS. These variables were previously found to be able to distinguish between younger and older adults (14) and between fallers and non-fallers (15). The single-step threshold was previously shown to be an independent predictor of a future fall (16–21) and to reflect unsteadiness in reactive balance control among older adults with varying histories of falls (15). The secondary laboratory outcome variables are foot contact time (voluntary stepping), and the ML CoP sway, as well as SDA parameters (Cd and Ds). All these parameters were previously found to reflect unsteadiness in balance control (13, 50, 55, 56, 58, 67, 68) related to falls (13, 55–58, 60), and also related to older adults who have bicycle riding habits (30). The other secondary outcome measures are the BBS, 6MWT, LLFDI, and FES-I. An intention to treat analysis is conducted by carrying the last obtained measurements forward for those participants who do not complete all aspects of the study. The significance level is *p* < 0.05.

The effect size (ES) (Hedge's g) between the two independent groups is calculated by dividing the difference between the means of each group by the pooled baseline standard deviation of both groups. Values between 0.2 and 0.49 are considered small, between 0.5 and 0.79 moderate, and 0.8 and higher is considered a large effect. In addition, for each outcome parameter, we explore whether a minimal detectable change has been reached. As data will be analyzed at the end of the study, there is no plan for interim analyses of primary and/or secondary variables.

### Adverse Events

Adverse events that meet all three of the following criteria will be reported immediately to the institution's Research Ethics Board, as is routine practice: (1) unexpected events in terms of nature, severity, or frequency; (2) events related or possibly related to participation in the research; or (3) events suggesting a potential increased risk of harm to research participants or others. All adverse events will be collected and evaluated bi-annually by the principal investigator.

### Data Monitoring

A data monitoring committee is not required for this study since the PBBT is a low-risk intervention for relatively healthy older adults without cognitive impairments. Based on our previous studies, very mild adverse events related to PBBT have been reported (i.e., delayed-onset muscle soreness, fatigue, or exacerbation of joint pain) in older adults and in people with stroke (28, 70–72), but medical attention was not required. with a similar frequency and severity of adverse events for both the PBBT and control groups, who completed more “traditional” physical therapy. Therefore, the typical adverse events are not specific to the PBBT.

### Feasibility Study

**Figure 5:**
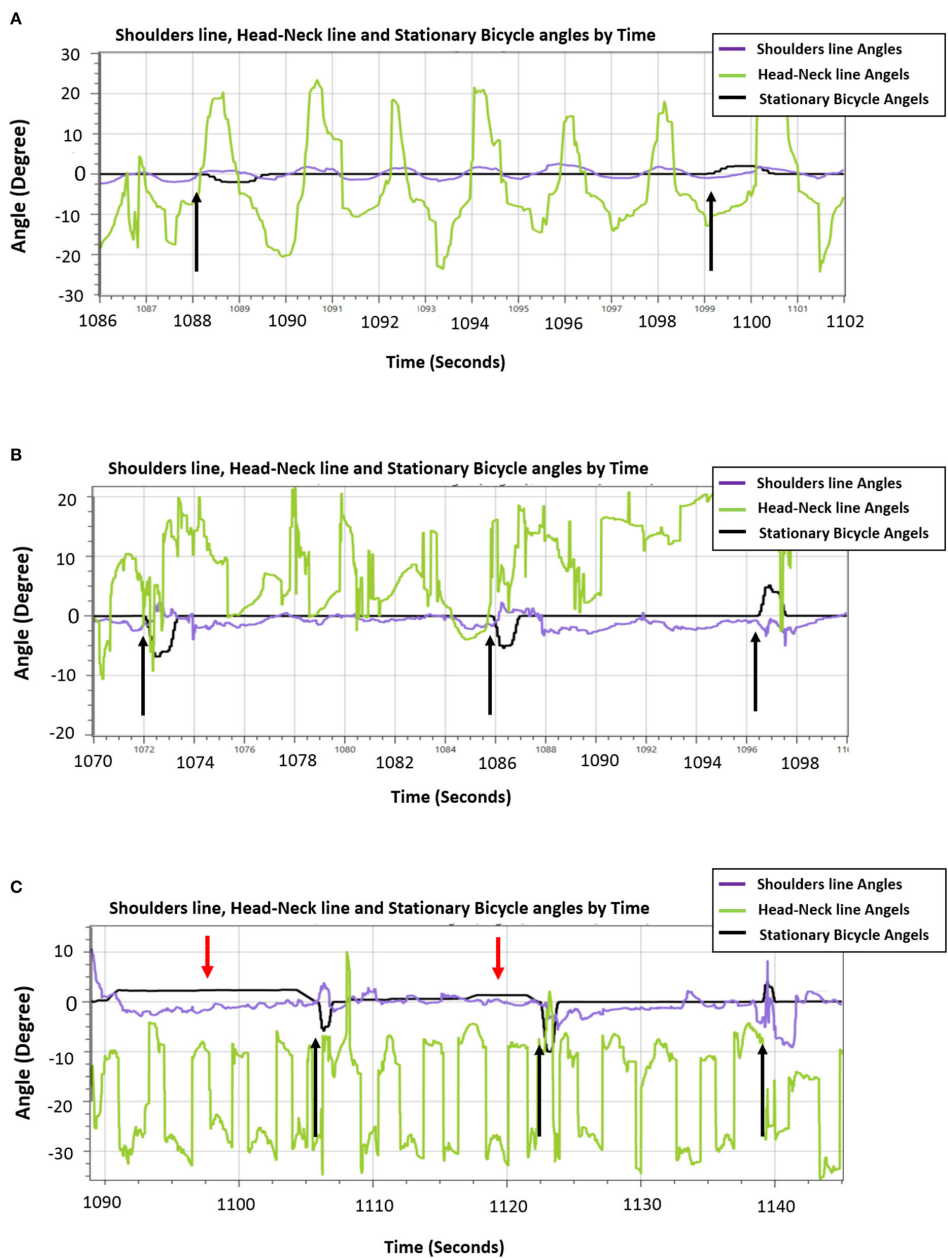
An example of the ability of the 86-years old trainee to reactively respond to unannounced perturbations during hands-free pedaling along the training sessions. **(A)** Low-magnitude perturbations in block right-left training of 2.5° tilt (i.e., the black arrows). A sample of 26 s that represents the participant's ability to consistently react to perturbations [shoulder line angles (purple line) reacts in the opposite direction and related to the black line perturbations]. **(B)** An example of effective trainee's reactive balance responses during random unexpected moderate-magnitude (6°-10° tilt) perturbation training. A sample of 30 s that represents generally organized and controlled shoulders/trunk movements [shoulder line angles (purple line] during hands-free pedaling, and particularly organized and effective upper-body balance responses (shoulders' response, represented by purple line rises in a manner adapted to the perturbations). **(C)** A sample of 60 s during the fourteenth session that represents the participant's ability to respond reactively to high-magnitude (8°-12° tilt) random unannounced external perturbations (spikes in the black line that represent the stationary bicycle training angles, black arrows) and respond proactively to self-induced perturbations during the “floating” mode of the moving platform (gentle long humps in stationary-bicycle-training black line, red arrows). Generally, the shoulders' balance responses show an organized response appropriate to the challenge of balance, i.e., the shoulders' purple line move in the opposite direction when the self-induced perturbations occurred and usually also in the case of unannounced external perturbations.

Here, we present results of a feasibility study to explore the ability of 86-years-old person to train and react effectively to unannounced perturbations during 14 training sessions of bicycling on PerStBiRo system with increasing perturbations magnitudes. The 86-years-old person reported few falls in the past 6 months, with high fear of falling. His upper-body balance reactive responses are presented by the shoulder line and head–neck angles. These parameters presence of an upper-body balance reactive response. The skill acquisition and motor learning of the 86-year-old male during the training is demonstrated in Figures 5A–C.

In Figure 5A an example from the 3rd training session where the participant was exposed to low magnitude, velocity and acceleration in low frequency of announced perturbations (2.5° tilt) in block right-left training during hands-free pedaling. Figure 5A shows that that despite pedaling without external support (hands free), participant reactively responded well when he exposed to unannounced balance perturbations. Note: The tilting perturbation evoked balance reactive trunk, head, and arm movements always in the opposite direction of the perturbation to quickly move the upper body's CoM toward the base of support provided by the stationary-bicycle seat (Figure 5A).

In the eighth training session (Figure 5B), the trainee was exposed to random moderate magnitudes, velocities, and accelerations of unannounced external perturbations (6°-10° tilt) during hands-free pedaling. Figure 5B shows an increase in the older person's ability to response reactively to perturbations of varying difficulty, and also that the PerStBiRo software are able to monitor and recognize reactive balance responses and to provide the trainee with sensorimotor feedback of his effective responses. Note, although this in the eighth training session, the magnitudes of perturbations were increased the trainee's balance reactive responses. The participant was capable of contract responding to with these challenges effectively, most likely probably due to his past experience.

In the fourteenth training session (Figure 5C), the participant was exposed to random high magnitudes, velocities and accelerations of unannounced external perturbations (8°-12° tilt) that were provided during hands-free pedaling in the unfixed and unstable mode of the moving platform (the “floating” mode). Figure 5C demonstrates that the proactive (indicated by red arrows) and reactive (indicated by black arrows) upper-body balance responses were appropriate most of the time for the diverse challenge of the perturbations, and therefore, reflect an effective motor learning of upper-body balance reactive responses that was acquired over the past training sessions.

This feasibility study helps to show that balance reactive responses can be evoked and improved by older adults and that the participant was able to train in an advanced training session (8th and 14th sessions) and still react effectively to higher levels of perturbations.

### Trial Status

The study is currently recruiting participants. Enrollment began on March 1, 2019. We will complete the recruitment, training, and T1 and T2 data collection by December 31, 2021. We will complete the data analysis by December 31, 2022.

## Discussion

### Strengths

This is a novel intervention method of a technology that provides self-induced and unexpected perturbations during stationary bicycle riding, which is designed to improve balance function during standing and walking among pre-frail and frail older adults. It touches on an important point in the field of fall prevention, as well as rehabilitation and motor learning principles—the ability to transfer compensatory balance reactions that are acquired in a sitting position into a target context of balance control performances in standing and walking. This study follows a strict single-blinded RCT design so that both intervention groups are trained by the same bicycle simulator system (i.e., PerStBiRo), and participants do not know which group they belong to. Both intervention groups will gain from a similar bicycling protocol consisting of endurance (pedaling intensity) and cognitive training components and, most likely, improve their function by the end of the training period. The experimental intervention consists of several exercise components with respect to motor learning, strength, endurance and, especially, balance control such as avoiding external support on the handlebars that reduces postural responses (42), increasing perturbation magnitudes to create a challenge, shifting from block to random perturbation training methods (43, 44), varied practice (43), shifting from a stable environment (simulator device is fixed) that provides external perturbations only to an unstable environment (unstable surface of the simulator device) that combines external and internal self-induced perturbations in varied training (45), giving external visual and sensorimotor cues that lead to improve intrinsic sensorimotor feedback (41). All these components provide the best possible motor learning implementation of reactive balance response in a sitting position and allow these exercises to be customized according to each subject's ability. They are challenging, but never dangerous. Our training is designed for a 12-week intervention period, with each training session lasting about 20 min, which is a similar time to conventional physical therapy training, so that in future clinical applications, it can be fit in a standard physical therapy session.

### Weaknesses

In this study, we aim to improve balance control and balance reactive components by tilting perturbations. However, these tilting perturbations during bicycle riding may not be similar enough to balance loss situations that cause falls among older adults; thus, they may not be specific. Because of the lack of specificity in this model, we may find no effects of the intervention. Perturbations in the experimental group are limited to the ML direction only, while hip balance strategy, which is one target of this training method, are triggered mainly by anterior–posterior movements. Trainers are trained to follow the pedaling intensity according to the training program to control this endurance component in the two intervention groups, but although the bicycle resistance is set equally, it might be difficult to monitor the speed of pedaling for each participant. This program is less challenging than the PBBT in walking and standing, but it is still possible for older adults to have difficulties in hands-free riding or to progress very slowly in the training program, which may not be enough to lead to improvement. Lastly, since the PBBT is a challenging training approach, there is a risk that older adults will stop participating in the program (drop out). In regard to our RCT study, there are disadvantages (limitations) to the convenience sampling that may weaken this study. The convenience sampling can lead to the under-representation or over-representation of particular groups within the sample. For example, old volunteers that commits to train 3 months are different from the general population of older adults. This undermines our ability to generalize our results to the general population of older adults. However, in our RCT the participant is randomly allocated to two groups thus a significant improvement in balance control system in one of the groups may truly represent a change in the balance function.

## Conclusion

The feasibility of this training concept has been tested in a pilot study on a 86-years -old person. The results indicate that older adults are able to respond effectively to increasing levels of unannounced perturbations during stationary bicycle riding. The participant also stated that he would recommend the program to his friends and family. A randomized controlled trial has been conducted for almost 2 years to investigate effects of the proposed training program on gait and balance function in independent older adults' volunteers. The intervention was started on March 2019 and till now we have recruited 42 older adults. twenty-six participants completed the intervention and another 10 are at the end of the training program these days. Till now one old person were dropout out from the PerTSBR group (due to First flare-up of osteoarthritis that was diagnosed during the intervention period), and no one from the TSBR control group. Five candidates immediately after the pre-test regretted and did not want to participate. Few participants reported adverse event of muscle soreness at the first sessions of the training program, and one person from the PerTSBR group, reported slight worsening of his chronic back pain after the 16th training session for the next few days. These effects were managed by adjusting the training intensity, and the symptoms disappeared during training. Compliance (attendance) to the exercise sessions were excellent (97% for PerTSBR group and 99% for the TSBR control group). Participants have reported that they felt safe while performing the exercises and found them challenging. Seven volunteers who completed the training program, are now in the training of the program in which they did not participate, outside of the research training program. We believe the proposed training technique merits additional study in future clinical intervention trials.

## Ethics Statement

The studies involving human participants were reviewed and approved by Soroka University Medical Center. The patients/participants provided their written informed consent to participate in this study. Written informed consent was obtained from the individual(s) for the publication of any potentially identifiable images or data included in this article.

## Author Contributions

IM and SB developed the intervention protocol and drafted the manuscript. IM, OL, SB, and YGB contributed to the study design and writing/editing the manuscript. All authors have read and approved the final manuscript.

## Conflict of Interest

SB and IM own a patent on some of the technology (PerStBiRo system) used in the perturbation system. We submitted a technical article describing the PerStBiRo system to BMC Geriatrics (no. BGTC-D-20-00476). The remaining authors declare that the research was conducted in the absence of any commercial or financial relationships that could be construed as a potential conflict of interest.

## Abbreviations

PBBT: Perturbation-based-balance training
BoS: Base of support
RCT: Randomized controlled trail
ML: Medio-lateral
PerTSBR: Perturbation training during stationary bicycling riding
TSBR: Training of stationary bicycle riding without perturbations
PerStBiRo: Perturbation stationary bicycle robotics
TV: Television
CoM: Center of mass
ICC: Intra-class correlation
AP: Anterior—Posterior
LLSS: Loaded leg side step
ULSS: Unloaded leg side step
COS: Crossover step
3D: Three-dimensional
EO: Eyes open
EC: Eyed closed
CoP: Center of pressure
SDA: Stabilogram-diffusion analysis
BBS: Berg Balance Scale
6MWT: Six-Minute Walk Test
LLFDI: Late Life Function and Disability Instrument
FES-I: Falls Efficacy Scale-International
MMSE: Mini Mental State Examination
ANOVA: Analysis of variance
SE: Effect size
Ct: Critical time (SDA parameter)
Cd: Critical displacement (SDA parameter)
Ds: Short-term effective diffusion coefficients (SDA parameter)
Dl: Long-term effective diffusion coefficients (SDA parameter).
