# Supplementary material for: The Effects of Bicycle Simulator Training on Anticipatory and Compensatory Postural Control in Older Adults: Study Protocol for a Single-Blind Randomized Controlled Trial
Source: Front Neurol. 2021 Jan 18;11:614664. doi: 10.3389/fneur.2020.614664 (PMC7848125; doi:10.3389/fneur.2020.614664)
Supplement: Supplementary file 2 [file Table_2.DOCX]

**Supplementary Table 1:** Administrative information

| **Data category** | **Information** |
| --- | --- |
| **Primary registry and trial identifying number** | ClinicalTrials.gov, NCT03636672 |
| **Date of registration in primary registry** | July 22, 2018 |
| **Date of study start** | March 1, 2019 |
| **Primary completion (Estimated date for completion of data collection)** | December 31, 2021 |
| **Study completion (Estimated date for completion of data analysis)** | December 31, 2022 |
| **Secondary identifying numbers** | BARZI0104 |
| **Source(s) of monetary or material support** | Helmsley Charitable Trust through the Agricultural, Biological, and Cognitive (ABC) Robotics Initiative and by the Marcus Endowment Fund both at Ben-Gurion University of the Negev. |
| **Primary sponsor** | Barzilai Medical Center, Ashkelon. Israel |
| **Secondary sponsor(s)** | \|  \|  \| \| --- \| --- \|   Itshak Melzer |
| **Contact for public queries** | Itshak Melzer, PhD [972-50-8807990] [itzikm@bgu.ac.il] |
| **Contact for scientific queries** | Itshak Melzer, PhD [972-50-8807990] [itzikm@bgu.ac.il] Ben-Gurion University of the Negev, Beer-Sheva, 84105, Israel. |
| **Location** | Ben-Gurion University of the Negev, Beer-Sheva, 84105, Israel. |
| **Public title** | Bicycle Simulator Training in Older Adults: A Randomized Controlled Trial (PerStBiRo) |
| **Scientific title** | The Effects of Bicycle Simulator Training on Anticipatory and Compensatory Postural Control in Older Adults: A Randomized Controlled Trial |
| **Countries of recruitment** | Israel |
| **Health condition(s) or problem(s) studied** | Accidental Falls |
| **Intervention(s)** | Procedure: Perturbation training  Experimental: Perturbation training during stationary bicycle riding  Active comparator (control): Stationary bicycle riding training |
| **Key inclusion and exclusion criteria** | Ages eligible for study: ≥70 years; Sexes eligible for study: All; Accepts healthy volunteers: Yes. Inclusion criteria: age 70 and over, able to walk independently, the provision of a medical certificate from a family physician allowing participation in physical training that requires walking two or three times a week. Exclusion criteria: Suffers from ischemic heart disease which restricts exercise, chronic obstructive pulmonary disease, and uncontrolled blood pressure; severe vision problems (blindness); mini-mental score of 24 or less; at least one year after hip or knee replacement surgery or after fractures of the lower extremities; amputation on the lower limb; neurological diseases or after a stroke. |
| **Study type** | Interventional (Clinical Trial); Allocation: randomized; Intervention model: Parallel assignment; Masking: Double (Participant, Outcomes Assessor); Primary purpose: Treatment |
| **Date of first enrolment** | March 1, 2019 |
| **Target sample size** | 68 |
| **Recruitment status** | Recruiting |
| **Primary outcome(s)** | Compensatory Step Execution tests during Standing and Walking [Time Frame: Change from baseline to 3 months later] |
| **Key secondary outcomes** | Postural Sway measure using force plate; Voluntary Step Execution Test; Berg Balance Scale; 6-minute walk test (6MWT); Late Life Function and Disability Instrument (LLFDI); Falls Efficacy Scale-International [Time Frame for all: Change from baseline to 3 months later] |
| **Protocol Version** | Issue Date: November 28, 2017; Protocol Amendment Number: 001; Author(s): M.D.; Omri Lubovsky, PhD Itshak Melzer, Shani Batcir. |
| **Study Record Versions** **on ClinicalTrials.gov** | August 16, 2018—Original  August 19, 2018—Changes in Eligibility, Study Description and Study Status (Clarification of abbreviations and Correction of typographical errors)  January 20, 2019—Changes in Study Status (updated the estimated study start date).  July 26, 2020—Changes in Recruitment Status, Study Status, Contacts/Locations, Outcome Measures and Study Identification (updated the study status for Recruiting, and actual study start date and estimated Primary and Study completion dates, and also Contact information). |
